# Abstracts from the Patient Classification Systems International (PCSI) Conference 2015, 2016 and 2017 (14-17 Oct 2015) The Hague, The Netherlands; (04-07 Oct 2016) Dublin, Ireland; (09-13 Oct 2017) Sydney, Australia

**DOI:** 10.1186/s12913-018-2940-1

**Published:** 2018-03-21

## A1 Malaysian Diagnosis-Related Group (MY-DRG): development of cost and service weights for radiological procedures at Universiti Kebangsaan Malaysia Medical Centre (UKMMC)

### Syed Aljunid, Roszita Ibrahim, Amrizal Muhd Nur, Nor Haty Hassan, Siti A. Zafirah

#### International Centre for Case-mix and Clinical Coding, National University of Malaysia, Cheras, Kuala Lumpur, Malaysia

##### **Correspondence:** Syed Aljunid (saljunid@gmail.com)

**Background**

Although case-mix is an important tool to estimate costs for DRGs, there is a lack of literature on using cost and service weights to establish costs for radiology procedures. Most studies concentrate on consumables’ and equipment costs and use step-down costing methods. Few studies employ activity-based methods to estimate radiology procedure costs, thereby failing to include equipment maintenance costs and specialized staff hours involved in producing radiology procedure results. Since 2002, the Universiti Kebangsaan Malaysia Medical Center (UKMMC) has used the Malaysian Diagnosis Related Group (MY-DRG) patient classification case-mix system to stratify disease severity, estimate costs, and enhance healthcare quality and efficiency. MY-DRG has a maximum of 1,250 DRG groups and is based on UNU-CBG grouper. This study assessed radiology services’ costs for each MY-DRG based on severity of illnesses.

**Materials and methods**

A cross-sectional study was conducted to impute radiology service costs using Activity-Based Costing. The study used a database of non-surgical patients admitted to UKMMC in 2011, and grouped all non-surgical cases discharged that year into MY-DRGs. Radiology costs for each MY-DRG were imputed; costing data trimmed using the Lower-three-Higher-three (L3H3) method; and radiology service weights calculated. The top 10 cases in the MY-DRG list were analyzed. Radiology procedures cost for each MY-DRG groups were imputed and mean total costs per episode of care estimated.

**Results**

The Department of Radiology conducted 121,221 radiology procedures in 2011. Of the 25,754 discharges, 16,173 (62.8%) were non-surgical and selected for this study. After trimming, 20 MY-DRGs had the highest radiology service weights; 6 MY-DRGs were from Central Nervous System Groups. The highest was MY-DRG G-4-26-I (Other Nervous System Disorders-Mild), radiology service weight 0.1899. Additional high-weight codes and service weights: MY-DRG N-4-10-I (Renal, Urinary Tract Neoplasm & Kidney Failure-Mild, 0.1642); MY-DRG G-4-25-I (Concussion –Mild, 0.1497); B-4-11- II (Hepatobiliary & Pancreas Neoplasms- Moderate,) and U-4-15-I (Other Ear, Nose, Mouth & Throat Disorders-Mild, 0.1366). Radiology services are a significant component in each MY-DRG group for UKMMC’s non-surgical cases, ranging from 8.1--19.0% of the total cost per episode of inpatient care.

**Conclusions**

Medical specialists should be learn about these findings so they can reduce unnecessary radiology procedures and improve healthcare efficiency. The same process should be applied to other disciplines (i.e., pediatric, surgical), to generate more-accurate cost data than conventional case-mix costing. The information offers a useful guide for healthcare providers and/or specialists seeking to reduce resource wastage and enhance service efficiency.

## A2 Readmissions in Germany: a new analytic approach beyond current DRG payment rules

### Marc Berlinguet^1^, Dorothee Assenmacher^2^, Andre Cools^2^, Raphael Graf^2^, Axel Bruns^2^

#### ^1^3M Health Information Systems (HIS), Wallingford, Connecticut, United States; ^2^3M Medica, Neuss, Germany

##### **Correspondence:** Marc Berlinguet (mberlinguet@mmm.com)

**Background**

Germany has specific DRG payment rules that apply to hospital readmissions for the same basic DRG; surgical readmissions when initial admissions were from the same Major Diagnostic Category’s (MDC) medical portion; and clearly-identified complications. These readmissions are grouped with the first encounter (i.e., admission) and not separately paid. (The DRG Catalogue also excludes some DRGs from these rules). This study examined whether these rules identify all potentially-preventable readmissions.

**Materials and methods**

All 2014 discharges from a group of German hospitals were selected, using 3M’s Medica benchmark dataset. Readmissions within 30 days to the same hospital as the original admission were included. 3M’s Potentially Preventable Readmissions (PPR) algorithm was used to analyze the resulting sample of 235,805 discharges and assess whether readmissions were potentially-preventable.

**Results**

Cases that were collapsed in single encounters were discarded. Standard DRG exclusions rules were applied, given difficulties in identifying potentially preventable readmissions in certain DRGs (i.e., metastatic/complex cancers, trauma, neonatology). After these steps, 166,195 discharges remained for 138,374 patients; the annual readmission rate was 1.17% (24,006 readmissions).

Chains were created for these discharges, consisting of either “only admission” (OA, no readmission occurred), or “initial admission” (IA) followed by one or more “readmissions” (RA). “Transfers to the same or other acute care hospital” (RT) were identified separately. A total of 162,380 admission chains resulted, with 3,696 RAs or RTs; average of 1.039 RAs or RTs within 30 days post-discharge. The average PPR rate was calculated (the ratio of IA divided by OA plus IA). A PPR rate of 2.19% resulted. Table 1 illustrates MDCs with above-average PPR rates.

**Conclusions**

This system identified additional potentially-preventable readmissions. Hospitals may benefit from an analytic approach that adjusts respective case-mix DRGs and complexity levels, and compares observed versus expected readmissions. Record reviews of individual cases (especially higher-than-expected rates of MDCs/DRGs) can help confirm readmissions were preventable rather than stemming from mapping, documentation, or coding issues.

**Table 1 (abstract A2).** MDCs with above average PPR rates

MDC	Total Admissions	Admissions at risk (OA+IA)	Chains with 1+ readmission	PPR Rate (%)
Blood and Blood Forming Organs	2,193	1,405	51	3.63%
Infectious and Parasitic Diseases	2,937	1,801	65	3.61%
Hepatobiliary System and Pancreas	8,459	5,843	197	3.37%
Kidney and Urinary Tract	13,309	9,279	277	2.99%
Circulatory System	23,296	18,752	529	2.82%
Respiratory System	21,585	12,755	355	2.78%
Digestive System	28,943	21,493	558	2.60%
Neoplastic Disorders	3,611	264	6	2.27%
Mental Diseases	693	445	10	2.25%

## A3 The implementation of an All Patient Refined (APR) DRG grouper in Portugal

### Claudia M. Borges, Nuno Amaro

#### Departamento de Gestão e Financiamento de PrestaçÃμes de Saúde (Health Services Management Department), Administração Central do Sistema de Saúde, IP (Health System Central Administration), Lisbon, Portugal

##### **Correspondence:** Claudia M. Borges (cborges@acss.min-saude.pt)

**Background**

Since 1989, Portugal has used the DRG patient classification system (PCS) for hospital analysis and financing for inpatient settings, ambulatory surgery, and some medical procedures. ICD-10-CM/PCS requires additional patient information, prompting implementation of an All Patient Refined (APR) DRG grouper in 2015. APR characterizes hospital outputs differently than the All Patient (AP) grouper used previously.

**Methods and materials**

To assess the transition’s impact, almost 3.5 million episodes in the national hospital database from 2012—2013 were grouped into AP DRG (version 27) and APR (version 31). Correlation tests were conducted on several variables (i.e., Length of Stay [LOS] Adjusted Index, Mortality Adjusted Index); financial impact, new production distribution, trim points, and relative weights were compared between systems.

**Results**

The APR DRG grouper has more homogenous groups and designations closer to clinical expertise than AP; it also subdivides episodes into severity and risk of mortality levels. A higher concentration of episodes results: 27.5% of inpatient cases group into 8 DRGs spread among severity levels, versus 22% grouped into 8 DRGs using AP.

APR groups 83% of episodes into low-severity levels 1 and 2. Half (52%) of episodes AP classifies as “complications and comorbidities” (CC) or “CC Major” group into APR severity levels 1-2. LOS typically increases with severity level (level 1 LOS = 4.5 days; level 4 LOS = 23.5 days), although there is no strong correlation between LOS and severity level. Some hospitals demonstrate strong positive correlations between diagnosis and procedure number and severity level. LOS Adjusted Index indicated some institutions had lower LOS, despite larger concentrations of high-severity-level patients.

Almost 88% of episodes grouped into risk of mortality levels 1 and 2; generally, mortality rates rise with increased risk of mortality level. Nevertheless, a weak positive correlation occurred between mortality rate and risk of mortality rate; some hospitals had negative correlations. Mortality Adjusted Index indicates some hospitals have better performance despite serving high-mortality-risk patients.

**Conclusions**

APR groupers require detailed information to split care into different severity and mortality risk levels. ICD-9-CM coding, while well-used by Portuguese National Health Service hospitals, may lack information on the patient process, which compromises adequate coding. APR groupers foster improvements in clinical record quality. Its concentration of episodes in severity levels 1-2 may also highlight hospital coding based on potential financial return, rather than care delivered. The APR complicates attempts to guess DRG results, incentivizing hospitals to focus on delivering high-quality care.

## A4 A new system to classify rehabilitation facilities’ outpatients for financing purposes

### Claudia M. Borges^1^, Elizabeth Reis^2^, Dália Nogueira^3^, José Dias^3^, Abdul Suleman^2^

#### ^1^Departamento de Gestão e Financiamento de PrestaçÃμes de Saúde (Health Services Management Department), Administração Central do Sistema de Saúde, IP (Health System Central Administration), Lisboa, Lisboa, Portugal; ^2^Instituto Universitário de Lisboa (ISCTE-IUL), Lisboa, Lisboa, Portugal; ^3^Escola Superior de de Saúde de Alcoitão (ESSA), Alcoitão, Alcabideche, Portugal

##### **Correspondence:** Claudia M. Borges (cborges@acss.min-saude.pt)

**Background**

Portuguese outpatient rehabilitation facilities are financed on a fee-for-service (FFS) basis, and do not differentiate by the complexity of patient disabilities. No systematic information is available about patients’ functional dependence, diagnostics, or demographic characteristics. Financing systems that do not address the amount of healthcare needed allocate resources inefficiently, since higher-dependency patients need more resources and rehabilitation time. Systems detached from patient case-mix and disease burden create inequities and incentivize prioritization of less-complex patients. Conversely, evidence-based financial resource allocation enables prospective systems based on patients’ clinical and functional status. Portugal’s Ministry of Health is studying development of an ambulatory rehabilitation financing system based on case-mixed-grouped function and complexity levels.

**Materials and methods**

A sample of patients was retrospectively classified, and patient dependency level measured using a shorter classification of the World Health Organization’s “International Classification of Functioning, Disability and Health” (ICF) developed for specific patient groups. This classification is a practical and efficient tool to classify and describe patient functioning. Gender, age, and diagnostics (i.e., ICD-10-CM) were also collected. Grade of membership (GoM) data representation was used to cluster patients and create a severity indicator for each patient. A Classification and Regression Trees model was applied to create different patient groups using this severity variable.

**Results**

The resulting 1,850 episodes were classified into Core Set 1 (neurological), 344 patients (18.6%); Core Set 2 (musculoskeletal), 1,404 (75.9%); and Core Set 3 (cardiopulmonary conditions), 102 (5.5%). The model resulted in 51 homogenous patient groups, divided into 14 impairment groups. Half (50.1%) of the patients were classified into the inflammation impairment group, resulting in a low total case-mix index (0,8663) for the studied sample. Only 1.5% of patients were grouped into non-progressive diseases of the nervous system, with a case-mix of 1.0943.

**Conclusions**

Ambulatory rehabilitation patients treated by the Portuguese National Health System have common characteristics in terms of severity and functional dependence, enabling complexity grouping. The most common patients are low-severity patients with musculoskeletal conditions, indicating that less-complex patients are more attractive in a FFS financing system. Creation of an Ambulatory Patient Classification system is the first step in implementing a prospective financing system. General practitioners are now measuring patient functional level, enabling patient ranking by complexity; in the future, the payment will be adjusted to patient complexity. Eventually, complexity will be a proxy for the care rehabilitation outpatients need, and financing adjusted to complexity.

## A5 The evolution of Activity-Based Management performance reporting in New South Wales – from top-down to predictive modeling

### Alfa D’Amato (alfa.damato@doh.health.nsw.gov.au)

#### New South Wales Ministry of Health, North Sydney, New South Wales, Australia

**Background**

New South Wales’ (NSW) Health Activity-Based Management (ABM) performance monitoring system has evolved over the last three years to meet the stakeholders’ and system manager’s requirements. Embedding ABM in the health system requires more up-to-date data and reports that can track each health service’s ABM performance against activities and costs. The ABM performance monitoring process is used to estimate, on a year-to-date basis, overall spending on activity-based funded (ABF) services and block funded services, provide variance against activity targets, and combine these results with overall financial results using a price-volume variance analysis. This process has been performed on a monthly basis for several years. Initially, calculations and reporting were performed in Excel-based templates based on top-down cost modeling. The evolved process now uses predictive modeling based on the last costing results applied to data on current year-to-date activities. The predictive modeling provides a state-wide view for system managers and enables business users to analyze cost information by service stream, classification, facilities, and/or specialty level.

**Methods and materials**

Cost was modeled using a Gamma regression, based on the most recent costing data. Gamma regression extracted and summarized numeric relationships between each cost driver (predictor), and estimates each cost driver’s contribution to the total cost. Using relationship estimates from the regression model has fostered the ability to predict the cost of new encounters.

**Results**

ABM is an evidence-based management approach that focuses on using patient-level data to inform strategic decision-making. Through clinical costing results and other activity data, ABM allows clinicians and managers to identify areas for improvement and make informed decisions about patient care by optimizing resource allocation. A system for continuous improvement, it provides a link with services’ Key Performance Indicators in which activity, cost, and performance information is used to achieve strategic and operational goals. ABM Performance Reporting aims to provide a strategic advantage to all stakeholders involved with case-mix, finance, and performance monitoring. This development has been embraced by the financial team and is now incorporated in the monthly reporting process.

**Conclusions**

Use of ABM has created an opportunity to connect the system’s key elements including finance, purchasing, and case-mix/ABF. ABM Performance Reporting’s use of predictive modeling has further strengthened this connection and fostered a transparent environment that succinctly monitors the health system’s performance based on price-volume variances. This process allows deeper understanding of the causes of the financial and activity variances on a year-to-date basis.

## A6 Implementing the Episode Clinical Complexity (ECC) model into the Australian refined Diagnosis-Related Groups classification for Version 8.0

### Vera Dimitropoulos (vera.dimitropoulos@sydney.edu.au)

#### National Centre for Classification in Health, University of Sydney, Sydney, New South Wales, Australia

**Background**

Phase one in the development of Australian Refined Diagnosis Related Groups (AR-DRG) Version 8.0 (V8.0) included reviewing the AR-DRG Classification Case Complexity Process. This resulted in a new Episode Clinical Complexity (ECC) Model, which allows an Episode Clinical Complexity Score (ECCS) to be assigned to each episode. The ECCS quantifies relative levels of resource utilization within each Adjacent Diagnosis Related Group (ADRG) and is used to split ADRGs into DRGs based on resource homogeneity.

**Methods and materials**

Derivation of an ECCS for each episode begins by assigning a Diagnosis Complexity Level (DCL) to each diagnosis in the episode. DCLs are integers between 0-5 that quantify resource utilization levels for each diagnosis, relative to levels within the episode’s ADRG. An algorithm combines the episode’s DCLs and defines its ECCS. This algorithm combines DCLs in descending order, and includes a decay component to adjust for multiple diagnoses’ diminishing contributions, vis-a-vis their individual contributions.

In Phase two, the ECC Model was implemented within AR-DRG classifications. A comprehensive set of ADRG splitting models was evaluated against classification structure principles, splitting criteria, statistical performance, and clinical relevance. Use of non-complexity splitting variables was minimized in favor of ADRG splits based on relative complexity (i.e. ECCS). As a result, only 6 of 403 (non-error) ADRGs require the use of a non-complexity splitting variable. The Classifications Clinical Advisory Group and DRG Technical Group (DTG) provided information on the proposed splits’ validity; further analysis on specific classification areas occurred before AR-DRG V8.0’s finalization.

**Results**

The AR-DRG classification structure itself was unaltered for V8.0, other than changes required by a surgical hierarchy review and minor code movements facilitated by incorporation of DTG-approved DRG public submissions. V8.0 has 807 DRGs (including 3 error DRGs). It demonstrates comparable statistical performance to V7.0 in ADRGs where length of stay (LOS) was removed as a splitting variable, and outperforms V7.0 in almost all other ADRGs where splitting occurred.

**Conclusions**

The conceptually-based, theoretically-derived, and data-driven characteristics of the ECC Model provides a strong basis for ongoing refinement of classification stemming from changes in clinical care and improvements in data quality. V8.0 represents a significant refinement to the AR-DRG classification, with major improvements in measurement of clinical complexity through the ECC Model, and greater transparency resulting from simplified splitting logic. These refinements will provide improved performance and support for the AR-DRG classification in roles that include hospital funding, health system analysis, and clinical management.

## A7 Patient segmentation for population health management using Clinical Risk Groups (CRGs)

### Herbert Fillmore (hhfillmoreiii@mmm.com)

#### 3M Health Information Systems (HIS), Troy, New York, United States

**Background**

Patient populations range from “non-users” to the critically ill, with degrees of complexity in between. These groups present different risk levels affecting financial and quality performance. Hence, hospitals benefit from identifying clinically-meaningful cohorts for targeted interventions that improve performance. This study applied a new aggregation of 3M Clinical Risk Groups (CRGs) to a population data analysis of clinical status’ financial impact.

**Methods and materials**

CRGs classify individuals’ health status and are derived from readily-available encounter and claims diagnostic information. CRGs differ from other case-mix systems by outputting mutually-exclusive classification groups that recognize and display interactions among conditions and severity of illness burdens. Because CRGs’ full array of 1,344 groups is too many for population health analysis, seven aggregated population health categories were created: “Non-user,” “Healthy,” “At-risk,” “Stable,” “Simple Chronic,” “Complex Chronic,” and “Critical.” Categories were applied to a blend of 2013 and 2014 data on over 2 million United States patients in three settings: a state-wide all-payer cohort; a commercial insurance plan; and a plan with predominantly Medicaid and Medicare beneficiaries (i.e., public plan). Individuals without at least 12 months of enrollment or with external costs of care (i.e., benefits coordination) were excluded. Relative percentages of members in each cohort and setting were compared with the cohort’s total costs of care.

**Results**

“Healthy” and “Non-users” represented 70% of the state-wide pool, 48% of the commercial plan, and 41% of the predominantly public plan. Neither used over 8% of total healthcare expenditures in their setting. Cohorts were smaller among more complex patients with “Critical” health status: 2% of the state-wide pool, 1% of the commercial plan, and 3% of the primarily public plan. And, total expenditures were significantly greater than cohort size: 13% of total expenditures for the state-wide pool, 12% for the commercial plan, 25% for the primarily public plan. “Simple Chronic” and “Complex Chronic” cohorts represented other expenditures disproportionate to cohort size.

**Conclusions**

Individuals with more complex disease burdens require care from multiple resources, including enhanced primary care teams, high-value specialists, and/or institutional care. Developing effective programs at the system level, and timely interventions at the individual level, are essential to delivering timely and efficient care. Reliable patient segmentation is key to achieving these goals. CRGs successfully differentiate among settings and populations, assisting in the design and delivery of appropriate healthcare. These data also help improve providers’ and payers’ understanding and acceptance of population differences.

## A8 Profiling high utilizers in social care and healthcare

### Tomi Malmström^1^, Antti Peltokorpi^2^, Markus Lappalainen^1^

#### ^1^Department of Industrial Engineering and Management, Aalto University, Aalto, Finland; ^2^Department of Civil and Structural Engineering, Aalto University, Espoo, Finland

##### **Correspondence:** Tomi Malmström (tomi.malmstrom@nhg.fi)

**Background**

Internationally, 10% of the population consumes over 70% of healthcare costs. This research explored service usage and characteristics of high utilization of social and healthcare services in a Finnish municipality (population 33,520) during 2011-2012. It assessed use of social care; broadened the “high utilization” definition to include service costs; and profiled different patient groups.

**Methods and materials**

Data repositories maintained by the National Institute for Health and Welfare (THL), which track a range of person-specific data for publicly-funded social and healthcare services, were used. Data capture most social welfare usage and all publicly-funded healthcare at an individual level. “High utilizers” (HU) were defined as the costliest 5%; “low utilizers” (LU) were defined as the least-expensive 95%. Each HU’s cost profile was evaluated.

**Results**

The costliest 5% of population incurred 65% of total costs; the costliest 10% consumed about 77% of total costs. Women used, on average, 0.2 more service categories and incurred slightly-higher average costs than men. When HUs were classified by most-expensive service category, average age/life stage was the most-distinctive feature. Average age by category:

- Child/Adolescent (age 0-18): “Child welfare”
- Adult (age 18-40): “Social assistance”
- Middle-Age (age 40-60): “Specialized somatic care,” “Specialized psychiatric care,” “Disability services,” “Mental health services”
- “Older people” (60+): “Primary care: inpatient,” “Services for older people.”

Table 1 presents category expenditure distributions.

**Conclusions**

An analysis of individual’s social and healthcare service use and definitions of HUs are both needed to identify underlying problems in patient episodes. Customer profiles in different high-utilizer groups vary significantly, suggesting that programs targeting specific populations and service organizations might curb high service use. The research provided new conceptual tools to identify and categorize HU populations.

**Table 1 (abstract A8).** HU and LU users’ service category use

2012 (total cost €, 81 million)	N	% HU	Average cost/ individual, €	% total costs	N	% LU	Average cost/individual, €	% total costs	
Child welfare	60	3.6	53,441	4.0	11	0.0	3381	0.0	5.46
Disability services	57	3.4	41,651	2.9	24	0.1	2499	0.1	2.38
Mental health services	235	14.0	15,174	4.4	1078	4.0	631	0.8	0.22
Primary care: Inpatient	743	44.3	13,113	12.1	586	2.2	2102	1.5	1.27
Services for older people	796	47.5	17,89	17.7	814	3.0	732	0.7	0.98
Social assistance	159	9.5	4,131	0.8	838	3.1	1877	2.0	0.19
Specialized psychiatric care	151	9.0	20,52	3.8	161	0.6	1448	0.3	0.94
Specialized somatic care	1145	68.3	10,759	15.3	7084	26.0	1493	13.1	0.16

## A9 Moving towards ICD-10 in Belgium at the expense of ICD-9 coded data?

### André J. Orban^1^, Luc B. Belmans^2^

#### ^1^Medical Department - Data Registration, AZ Alma, Eeklo, Belgium; ^2^Medical Department - Direction, RZ Heilig Hart Tienen, Tienen, Belgium

##### **Correspondence:** André J. Orban (andre@caphoda.be)

**Background**

Belgium transitioned from ICD-9-CM to ICD-10-CM and ICD-10-PCS (called ICD-10-BE) on January 1, 2015. To achieve this migration, the Federal Public Service of Health, which is responsible for collecting the Minimal Hospital Discharge Data Set (MHDDS), encouraged hospitals to submit their data on time. Healthcare institutions were simultaneously training coders in ICD-10, and completing an above-average workload to complete the final time-periods coded in ICD-9. This study analyzed the extent to which these internal and external factors impacted the quality of the last data sets coded in ICD-9-CM.

**Methods and materials**

The study reviewed medical and nursing data on inpatient stays with a 2012-2014 discharge date provided voluntarily by hospitals in accordance with the typical Belgian MHDDS standard. The study used an automated alternative to audit coding quality. Previously-developed “coding alerts” (a set of queries based on the existing Belgian coding guidelines for ICD-9-CM v.2011) were re-used and enhanced. The study then measured concordance between medical diagnostics and procedures provided with nursing activity elements. The researchers assumed that nursing data were unaffected by the ICD-10 transition, since they are registered in most hospitals by a separate coding team. Hence, changes in nursing data after ICD-10-BE transition could be compared to the changes in medical data quality. A brief questionnaire was also conducted with participants to assess the impact of increased time pressure and preparations to migrate to ICD-10-BE.

**Results**

The overall ratio of stays triggered with at least one coding alert is low. One participant shows a slightly significant increase in alerts triggered over the last observed periods. More results are being discussed and individual reports and benchmark data being provided to participants. It is thought that coding teams gained a better theoretical understanding of anatomy and physiopathology during the transition, which could benefit the ICD-9 coding outcomes.

**Conclusions**

Belgium’s transition to ICD-10-BE required more detailed patient records, enhanced coding accuracy, and increased use of the MHDDS for quality and outcome purposes. The study’s review of final data submitted at the end of the old system’s use suggests that shifting to new coding systems may have a greater-than-expected impact on hospital data quality.

Coding alerts and medical/nursing-matches now need to be adapted for use with ICD-10-BE.

## A10 The U.S. Medicare program’s quest to obtain value for money spent: tying case-mix payments to performance, quality, and efficiency

### Jugna J. Shah (jugna@nimitt.com)

#### Nimitt Consulting, Inc., Spicer, Minnesota, United States

**Background**

By the end of 2018, the United States (U.S.) Department of Health and Human Services intends that almost all Medicare payments be tied to quality initiatives and/or Alternate Payment Models (APMs). Multiple initiatives are underway to incentivize hospitals and physicians to provide higher-quality, lower-cost healthcare without compromising outcomes. New payment initiatives are being investigated to reimburse facilities and physicians across a variety of care settings (i.e., inpatient, outpatient/ambulatory, day surgery, physician office).

Medicare will use and/or pilot-test several methods from 2016-2018:

- Collecting quality measures,
- Reducing payments for poor-performing hospitals on Hospital-Acquired Conditions
- Reducing payments for excessive hospital readmissions,
- Creating bundled care payment initiatives, including comprehensive joint bundle payments for elective hip and knee replacements,
- Value-Based Purchasing.

Some initiatives place a percentage of hospital MS-DRG reimbursement at-risk if facilities fail to achieve specific performance thresholds, and increase or decrease case-mix level payments based on performance. The latest initiatives that are being tested focus on paying for larger and larger bundles of services at a single rate (i.e., episode of care payment, flat rate for integrating care across sites of service, etc.).

**Results**

Specific measures and calculations used in the payment initiatives were described to inform stakeholders about Medicare’s activities. These initiatives seek to link payment with specific outcomes metrics in order to transition the use of DRGs and APCs from a transactional payment model to a more dynamic payment tool. Leveraging existing claims and cost data is key to converting existing traditional case-mix-based payment systems and/or contracting models into those that enhance value for patients and reduce costs for patients, providers, and payers. In terms of quality initiatives, Medicare is moving away from process measures and focusing more on paying for high-quality healthcare services assessed via outcomes, patient experience, and efficiency measures.

**Conclusions**

It is difficult to know whether the U.S. is achieving better value for the money it spends. This is the goal of new value-based approaches, which hold providers more accountable for the care provided and outcomes achieved. These approaches measure providers against themselves and their peers on both improvements and achievements; place traditional case-mix dollars at-risk for low- and/or poor-performing hospitals; and reward high-performing providers. Ultimately, the new bundled care and episode initiatives seek to transform Medicare from a passive payer to an active purchaser of healthcare services.

## A11 Building a population grouping methodology

### Jeff Hatcher, Heather Richards, Craig Homan, Victoria Zhu

#### Canadian Institute for Health Information (CIHI), Ottawa, Ontario, Canada

##### **Correspondence:** Jeff Hatcher (JHatcher@cihi.ca)

**Background**

In 2013, the Canadian Institute for Health Information (CIHI) initiated development of the first population grouping methodology (a case-mix classification with predictive indicators of morbidity burden) and software using Canadian data, including all public Medicare beneficiaries.

**Methods and materials**

An additive classification with over 200 health-condition categories and mutually-exclusive classifications was developed. Health-condition categories were created and vetted by expert advisory groups; patient-level clinical and financial information was gathered and analyzed. Tagging rules ensured proper validation of diagnoses from physician billing data, which were used to capture visits to family medicine and specialist physicians.

Retrospective and prospective cost weights were produced for each registered person in the population. Linear regression models using an ordinary least squares estimation method were employed in fitting the models. Separate models were developed for three patient populations: health system non-users, users without health conditions, and users with health conditions.

Using the health condition categories and the most influential two-way health condition interactions, retrospective and prospective cost weights were predicted for users with health conditions. Models for non-users and users without conditions were developed using age and sex as the predictor variables.

Health conditions were rolled-up into groups of mutually-exclusive classifications, and individuals were tagged to their highest-ranked condition group. The groups were linked to higher-level categories that differentiated between chronic vs. acute conditions, severity of diagnoses, etc. Each cell was tested to determine various comorbidities’ effects on cost. Comorbidity splits provided a substantive increase in the grouper’s explanatory power and increased the number of cells.

**Results**

The methodology’s additive classification reflected total healthcare cost variations based on patient morbidity. Both models had satisfactory explanatory power. Goodness-of-fit analysis on validation data demonstrated the predictive models were stable and did not overfit to the estimation data.

**Conclusions**

Feedback indicates the development project was successful. Future work will refine and enhance both additive and mutually-exclusive methodologies and the first release of the project is scheduled for 2016. As additional clinical and cost data are incorporated and as additional predictive indicators are developed, future version of the methodology will also be released.

## A12 Does HRG4+ appropriately accommodate frail elderly patients in an acute care setting?

### Jill Cockrill (j.cockrill@hscic.gov.uk)

#### National Casemix Office, National Health Service Health and Social Care Information Centre, Leeds, West Yorkshire, United Kingdom

**Background**

The aging population is among the biggest challenges facing England’s National Health Service (NHS): two-thirds of hospital admissions are over 65; over one-quarter of inpatients have dementia. Frailty is not a formal diagnosis identified by a specific code, so it’s challenging to define and identify the “frail elderly.” Five identifiable conditions help define “Frailty Syndrome:” falls, immobility, delirium/dementia, incontinence, and polypharmacy. The NHS uses Healthcare Resource Groups (HRGs) developed and maintained by the National Casemix Office. The latest version, HRG4+, includes interactive “complications and comorbidities” (CC) based on summed scores of all secondary diagnoses in a patient record, which can differentiate frail elderly patients’ resource use.

**Methods and materials**

The study used Casemix Hospital Episode Statistics (CHES) data from 2014-2015 at Finished Consultant Episode (FCE) level; HRGs were derived from the HRG4+ 2014-2015 NHS Reference Cost design. A unit cost per FCE was calculated using Reference Cost provider-level source data by mapping Hospital Code, admission type, and HRG to corresponding CHES records. Unit cost included all of the core HRG’s costs of care, excluding unbundled HRG costs. Adjusted Length of Stay (LOS) was the episode duration. The published national average cost was used when no provider-level match was found.

The investigated HRG root, *LA04 Kidney or Urinary Tract Infections, without intervention*, is high-volume, has interactive CC splits, and is common among admissions for patients over 65. ICD-10 diagnosis codes that identify Frailty Syndrome conditions were flagged; each of the FCE record’s 20 ICD-10 code fields was assessed against the Frailty Syndrome categories; and the sum of unique frailty syndromes calculated (e.g., patient diagnosis relating to immobility, dementia/delirium and polypharmacy scores three). A Casemix Frailty Index (CFI) score was calculated for each FCE: *None*=0, *Mild*=1, *Moderate*=2, *Severe*=3, *Profound*=4+.

**Results**

Initial findings based on unplanned FCEs show frailty’s proportion and severity increases as HRG complexity increases. FCEs for patients with some frailty have higher average LOS within each HRG, but there are no significant cost differences between CFI categories for HRGs.

**Conclusions**

HRG4+ enhancements appear to accommodate frail elderly patients’ resource use when comparing CFI and HRG-level average costs. Findings are based on a small number of HRGs from one clinical area over one financial year. Further work is needed to investigate whether some subchapters accommodate frailty better than others. Establishment of a robust CFI creates opportunities including flagging patients for service planning, redesigning care packages, and updating reimbursement systems.

## A13 What information should a hospital board receive about hospital quality?

### Stephen Duckett (stephen.duckett@grattan.edu.au)

#### Health Program, Grattan Institute, Carlton, Victoria, Australia

**Background**

A 2012 survey of hospital boards in the state of Victoria found a curious phenomenon: virtually all respondents believed that the overall safety and quality of the care delivered at their health service was as good as, or better than, the typical Victorian health service. This mathematical impossibility suggests that many hospitals board members do not actually know how the care that is provided at their facility compares with other hospitals’ performance. Further, this indicates that the information the board receives from the hospital management is inadequate.

**Methods and materials**

As part of a government-commissioned review of the governance of safety and quality in hospitals in the state of Victoria, a standard “board report” was developed using routine data.

**Results**

Approximately 70 additional quality and safety indicators are proposed for use in reports to hospital boards. These indicators draw on indicator development generally in Australia, and specifically in Queensland. The proposed indicators include trend data on key indicators that are presented as statistical process control charts as used in Queensland, and data on the Australian Commission on Safety and Quality in Health Care’s “high priority complications.”

**Conclusions**

Boards need robust information on hospital performance in order to exercise effective oversight over their hospitals and to hold Chief Executive Officers to account. The proposed board report should increase board members’ understanding of safety and quality issues. The second group of indicators (i.e., high priority complications) will be a particularly helpful development for smaller hospitals.

It is recommend that such reports have key recommendations distilled on the first page, with information relevant to the hospital’s core business. Reports should be comprehensible to any board member, regardless of their clinical and/or statistical background.

## A14 Using routine data to measure “hotspots” of potentially preventable admissions

### Stephen Duckett (stephen.duckett@grattan.edu.au)

#### Health Program, Grattan Institute, Melbourne, Victoria, Australia

**Background**

The study sought to identify localities with high rates of potentially-preventable hospitalizations, called “ambulatory care sensitive conditions” (ACSCs). ACSCs were developed in the U.S. as prevention quality indicators to measure quality of out-of-hospital care. ACSC hospitalizations are often used as a proxy measure for primary care effectiveness and/or access to care.

**Methods and materials**

The 22 ACSC categories recognized by Australia’s National Healthcare Agreement are used as indicators of reducible health inequalities and potentially-reducible hospitalizations. The study calculated age-sex adjusted rates for 9 high-volume ACSCs and a 10^th^ combined measure of Chronic ACSCs over a decade, for areas with populations of 1,000 or more. The state-wide annual rate for each ACSC was the benchmark. Age-sex adjusted rates were used to divide ACSC hotspots into the following types, and identify where intervention is needed:

- *Hot enough:* An area with a rate of 50 percent or more over the state average for one or more ACSC.
- *Persistently hot:* Chance can generate high rates, so identifying persistently hot areas helps allocate resources to the best areas for intervention.
- *Likely to stay hot (predictable)*: Health interventions take time to develop, be implemented, and succeed. Current data is used to identify areas likely to be hotspots in the future, when interventions are in effect.
- *High impact*: Hotspots must have big enough health and/or financial impacts to warrant action. The potential impact of any action depends on several factors: number of individuals affected, severity of the condition, efficiency gains by targeting high concentrations of at-risk individuals, and equity gains. These must be balanced against the costs in order to evaluate whether to implement any interventions.

**Results**

Thirty-eight (38) priority places in Queensland and 25 in Victoria were identified with potentially-preventable hospitalization rates at least 50 percent higher than state averages in every year for a decade.

**Conclusions**

Intervention’s cost-effectiveness must be established on a small scale before implementation in other areas. A three-to-five year intervention trial in a small number of areas is recommended, using locally-developed place-based interventions that are rigorously evaluated.

An estimated direct savings of at least A$10-15 million annually could be achieved by reducing potentially-preventable hospitalizations to average levels; indirect savings should be significantly larger. Improved health will reduce healthcare costs, and improve well-being, opportunity, social cohesion, workforce participation, and productivity. Options for specific responses in priority places were also presented.

## A15 An Australian activity-based funding classification for teaching, training, and research

### Joanne Fitzgerald (joanne.fitzgerald@ihpa.gov.au)

#### Classifications and Coding Standards, Independent Hospital Pricing Authority, Darlinghurst, New South Wales, Australia

**Background**

Activity-based funding classifications are available for many types of patient care. This study assessed whether hospital teaching, training, and research (TTR) activities could be classified and funded on an activity basis. Results from a three-year Australian program indicate that classification for hospital-delivered teaching and training is feasible. Following the development of definitions of “teaching,” “training,” and “research,” and an initial exploratory cost driver analysis, in-depth data collection was undertaken. This TTR costing study, conducted at a sample of Australian hospitals, collected comprehensive activity and cost data to inform TTR classification development.

**Methods and materials**

A representative sample of health services was recruited to participate in the study; 19 sites were selected from metropolitan, regional, and rural locations in Queensland, Western Australia, and South Australia. Data were collected for a range of clinical teaching and training modes to derive comparative training costs for each type of trainee. Trainee and trainer-related costs were estimated for direct teaching and training activities; teaching and training delivered in conjunction with patient care; and other teaching and training support functions. The study also captured hospitals’ costs for supporting research capability. Health professionals who acquired a particular set of clinical skills were the TTR’s “output.” The study quantified public hospitals’ contributions towards skills acquisition with respect to the average cost per trainee full-time equivalent (FTE) who received teaching and training.

**Results**

The teaching and training component identified relative differences in costs for each type of trainee. On a fully-absorbed annualized cost basis, the average cost of training, per FTE, across all professions was approximately $48,480 annually for each profession. Trainees with the highest costs were:

- Medicine, advanced vocational: $70,104
- Dentistry, advanced vocational: $68,496
- Nursing and midwifery, early graduate: $63,012
- Allied health, advanced vocational: $38,868

Teaching and training delivered in conjunction with patient care consumed the highest component of the cost per FTE, often representing over 80% of these cost. Determining a suitable “output” to cost was problematic for site-based research; variations in costs were too significant to draw an adequate conclusion about representative costs across hospitals.

**Conclusions**

The TTR costing study’s results demonstrated the feasibility of identifying and costing a teaching and training product. This project demonstrated that it might be possible to cost research capability, but did not identify a relationship between research capability costs and research outputs. Consequently, a research product to support classification development for research was not identified.

## A16 Integrated neonatal care with appropriate funding and its implementation

### Jacob Hofdijk, Nienke Bults

#### Innovation, Casemix – CasemixQ-ConsultTalent (CQT) Group, Utrecht, Netherlands

##### **Correspondence:** Jacob Hofdijk (jhofdijk@gmail.com)

**Background**

The Dutch perinatal care system formerly distinguished between gynecologists addressing pathological aspects of pregnancy and childbirth, and midwifes providing physiological care. A statistical report indicated that the Netherlands had among Europe’s highest infant mortality rates prompted changes to this system. Steering group recommendations for improving Dutch perinatal care included formation of professional networks to deliver the best, safest maternity care. Implementing this recommendation necessitated creating agreed-upon perinatal care standards, and binding agreements about quality, registration, responsibility, and transparency. The integrated perinatal care approach also required creating episode-based payments that integrated Dutch primary and secondary care providers.

**Methods and materials**

The 2011 founding of the College of Perinatal Care (CPZ) initiated this system change; CPZ’s mission is to re-organize perinatal care, create the care standard, and implement it. The CPZ engaged in a successful process to improve collaborations between perinatal care providers in over 80 regions. It also developed care pathways describing new collaborations between midwifes, gynecologists, and maternity nurses. The care standard required formalizing all providers’ shared responsibilities and determining funding mechanisms that shifted away from traditional, institutional-based funding. The process generated major debates, during which, 20 regions continued elaborating the new integrated model and agreeing on new care processes.

**Results**

In the Hoorn region, case-mix was used to implement integrated perinatal care systems. The design principles underlying the system combined the existing guidelines and protocols from the perspective of mother and child, and translated them into a regional program with an embedded information standard. This created the basis for the individual care plan for mother and child, supported by an IT system that links to individual providers’ systems and is available to the mother. It provides financial services both for billing and paying the collaborating perinatal care providers their appropriate share.

**Conclusions**

Introduction of the integrated perinatal care system fostered a shift to person-centered care and focus on demand vs. supply. Mutual respect between providers and their association was key to successfully reorganizing perinatal care. The creation of the shared record used the principles of technological, semantic, and social interoperability. These formed the basis of individual care plans that balance patients’ conditions and life targets, a dimension of the Blue Line Connectivity standard that was applied to support collaborations between providers and patients. This approach may be applied to other multidisciplinary care approaches.

## A17 CHADx+: Extensions to the Australian classification of hospital-acquired diagnoses improve its utility in quality improvement

### Terri Jackson^1,2^, Amanda Ling^3^, Jennie Shephear^4^, Daniel Borovnicar^5^, Stuart Swain^6^, Peter McNair^5^

#### ^1^Population Health, University of Melbourne, Melbourne, Victoria, Australia; ^2^Melbourne Institute for Applied Economic and Social Research, University of Melbourne, Melbourne, Victoria, Australia; ^3^Ramsay Health Care, Joondalup Health Campus, Perth, Western Australia, Australia; ^4^Victorian Agency for Health Information, Melbourne, Victoria, Australia; ^5^Victorian Department of Health and Human Services, Melbourne, Victoria, Australia; ^6^HealthBench Pty Ltd, Perth, Western Australia, Australia

##### **Correspondence:** Terri Jackson (terri.jackson@unimelb.edu.au)

**Background**

The Classification of Hospital-Acquired Diagnoses (CHADx) system is increasingly used as a quality improvement tool in Australia. This use has highlighted the need for changes to the classification, reported here as the CHADx+ version.

**Methods and materials**

A code-by-code review of all International Statistical Classification of Diseases and Related Health Problems (ICD) codes used in the original CHADx was conducted. In addition, a review of all codes in the Australian Classification of Healthcare Interventions (ACHI) was undertaken to identify procedures that indicate remediation of in-hospital complications, and diagnosis codes in readmission episodes were reviewed to identify those associated with 30-day readmission.

**Results**

The changes made to Version 1 of CHADx have simplified the assignment rules in order to reduce the need for linkage between diagnosis codes; grouped all hospital-acquired infections into a single Major CHADx class; and split some classes to better distinguish serious complications from less-serious ones. 134 codes have been moved to the data-cleaning algorithm developed for the original version.

A second new procedures module (CHAPx) has been added to capture information on additional, non-principal procedures. This module enables the addition of information (i.e., a diagnosis of hemorrhage requiring transfusion) or identification of second surgical procedures undertaken due to complications in the episode (i.e., unplanned hysterectomy to manage intra- or post-partum hemorrhage).

A third module (Readmission Related CHADx (RR-CHADx) was developed that uses linked data across the state of Victoria to identify readmissions that may be attributable to complications from the original (index) admission.

Reports from these three modules have been incorporated into an online portal for use by the Victorian state Health Department, individual hospitals, and hospital-based clinicians to access comparative CHADx+ reports.

**Conclusions**

Classifications must constantly evolve to keep pace with medical innovation and new data uses. Access to local, timely, comparative data is essential to support clinical efforts to improve hospital care.

## A18 So, we have our CHADx data. What happens next?

### Jenny McNamee^1^, Michael Navakatikyan^2^

#### ^1^National Casemix and Classification Centre, University of Wollongong, Sydney, New South Wales, Australia; ^2^University of Sydney, Sydney, New South Wales, Australia

##### **Correspondence:** Jenny McNamee (jmcnamee@uow.edu.au)

**Background**

Australia is introducing funding incentives around hospital care safety and quality. The Classification of Hospital Acquired Diagnoses (CHADx) includes classes and sub-classes of ICD-10-AM diagnosis codes providing valuable information on adverse outcomes in hospital care and fostering practice change. Hospital Acquired Conditions (HAC) is a newly-defined, more targeted, set of adverse outcomes that are considered both serious and avoidable.

**Methods and materials**

To understand CHADx’s real costs and Length of Stay (LOS) impacts, a statistical model was developed using morbidity and cost profiles of hospitals in one New South Wales (NSW) Local Health District (LHD). An algorithm incorporating the CHADx code subset and condition onset flag was applied to a linked inpatient morbidity and cost dataset. Episodes with CHADx diagnoses were identified; same-day cases were excluded. The model was based on CHADx code number (vs. type), acknowledging potential interactions between complicating diagnoses versus treating them as independent events. The model adjusted for factors affecting cost and LOS (i.e., age, hospital-specific case-mix, episode acuity/urgency, pre-existing comorbidity levels). It was based on one year of data and tested on a second year of data.

**Results**

On average, an 18-19% increase in overnight episodes’ direct costs was attributable to CHADx. The largest relative cost increase was seen with the first CHADx code, followed by smaller (but equal) cost increases for subsequent CHADx codes.

To explore risk assessment and harm reduction through clinical engagement, a research collaboration was established with a second NSW LHD, to foster clinical practice change and monitor results. This collaboration sought to develop risk-assessment systems and data dissemination to: identify key risks and priority concerns about volume, cost, and patient flow; use multiple data sources to develop more-complete safety and quality assessments; and engage clinicians in practice change. The collaboration applies the same principles developed for CHADx to HAC data to estimate HACs’ impact and identify the LHD’s riskiest areas. For selected risk areas, a reporting methodology will be developed using morbidity, near-miss, and incident response data. Greater clinician engagement is expected to occur from using data clinicians created themselves.

**Conclusions**

Using multiple clinical and administrative data sources enhances understanding of risk drivers, safety, and quality. It engages clinicians in initiatives to reduce patient harms. Future work will identify patient demographics and clinical profiles indicative of high risk, and help distinguish avoidable versus unavoidable adverse event risks. Risk profiles should enable targeted, proactive interventions to reduce the HAC rates.

## A19 Using Time-driven Activity-based Costing (TDABC) to cost complex chronic illness: the case of dementia

### Daniel Regan^1^, Patrick Slevin^2^, Gerardine Doyle^3^

#### ^1^School of Psychology, University College Dublin, Dublin, Ireland; ^2^The Insight Centre for Data Analytics, University College Dublin, Dublin, Ireland; ^3^College of Business, University College Dublin, Dublin, Ireland

##### **Correspondence:** Daniel Regan (daniel.regan@ucd.ie); Gerardine Doyle (gerardine.doyle@ucd.ie)

**Background**

The term “dementia” describes a range of conditions that affect memory, thinking, language, and performance of everyday tasks. There are approximately 41,470 persons with dementia (PwD) in Ireland, with an estimated annual cost of approximately €1.69 billion and average annual cost per PwD of approximately €40,500.

Due to fragmented care and the lack of coherent Dementia Care Pathways (DCP), costing studies to-date have been limited by a lack of detail on how costs were generated at the patient level, and standardized costing protocol. A recent Irish National Dementia Audit found that 33 of 35 (94%) of relevant Irish hospitals had no DCP in place; implementation of a local DCP in each acute hospital was recommended. This highlights the need to develop an accurate care pathway onto which accurate, patient-level costs could be mapped.

**Methods and materials**

The study used “time-driven activity-based costing” (TDABC), a costing approach that uses methods from accounting and social sciences to identify and cost DCPs. Due to dementia’s complex nature, vignette-based surveying was used to facilitate accurate, valid costing. Two patient exemplars (i.e., clinical case scenarios) with distinct dementia profiles were developed, representative of the majority (70%) of “typical” dementia cases. Through a combination of 140 hours of participant observations, and over 105 semi-structured interviews, a cost for “typical” DCPs through the Irish public health system was derived. The methodology provided information across all discrete stages along the DCP.

**Results**

The results confirmed that dementia is an extremely costly condition at both individual and societal levels, and that costs of care increase considerably with the condition’s severity and duration. For the comprehensive exemplar cases, monthly costs for dementia’s four stages were: *mild* (€2,640-€3,713); *mild-to-moderate* (€3,291-€6,083); *moderate-to-severe* (€3,361-€6,808); and *severe* (€7,614). Ireland’s estimated average annual care costs were: €387,232,495; €285,692,492; €221,400,985; and €730,015,878, respectively—approximately €1.624 billion in annual health service costs.

**Conclusions**

The study provided the first in-depth patient-level mapping and costing of dementia care. It is an important first step in generating detailed, valid information about integrated care provision’s costs. If costs for all remaining dementia cases were equal, Ireland’s true dementia cost is around €2.32 billion annually, considerably higher than the €1.69 billion previously estimated. Since many cases are more complex (e.g., Lewy Body dementia) and more expensive than the study’s two samples, actual costs are likely to be higher. TDABC and vignette-based surveying are recommended as useful ways to cost complex conditions.

## A20 Is case-mix adjustment in primary care at the mercy of coding quality? Yes … and no

### David Shepherd^1^, Mark Pierce^1^, James Barrett^2^, Alan J. Thompson^2^

#### ^1^Leicester City Clinical Commissioning Group, Leicester, United Kingdom; ^2^Johns Hopkins Healthcare Solutions, Baltimore, Maryland, United States

##### **Correspondence:** Alan J. Thompson (athompson@HopkinsACG.org)

**Background**

Primary care clinicians know that case-mix varies between different physician practices and geographical areas. Leicester City Clinical Commissioning Group (CCG) is now able to quantify those differences using routinely-collected primary care and hospital data that are grouped and processed by the Johns Hopkins Adjusted Clinical Groups (ACG®) System. Quantification of relative case-mix across a range of practices is facilitating new types of analyses of practices and performance data. However, it is important to understand the impact of data quality and completeness to ensure these analyses are robust.

**Methods and materials**

Johns Hopkins personnel worked with Leicester City CCG to validate the ACG System’s case-mix-related outputs and evaluate their usefulness to improve primary healthcare quality, equity, and performance. Two metrics were examined: secondary care costs and emergency admission rates. One concern was whether variation in coding quality and completeness negatively affects the validity of case-mix-adjusted outputs. Examining “standardized morbidity ratios” across diagnostic groups confirmed significant coding variation across the CCG’s practices. A computer model was developed to quantify the level of under-coding by practices and further adjust the case-mix-adjusted outputs to take this variation into account.

**Results**

Results illustrate the variation between *expected* cost and activity levels (adjusted for case-mix) and *observed* levels. This allows practices to be compared with their expected levels rather than the average for the CCG. The results also showed the degree of variation attributable to coding quality and its impact on the case-mix-adjusted data. Despite coding quality issues, it was possible to identify the main outliers with regard to higher-than-expected healthcare utilization levels and to identify those practices performing better than expected.

**Conclusions**

The ACG® System can be used to case-mix adjust healthcare utilization levels based on morbidity to enable meaningful comparison across physician practices. A computer model was successfully developed to adjust for any local under-coding to create robust *observed* versus *expected* levels of activity and performance. Where observed costs or emergency admission rates are *lower* than expected in a practice, commissioners can investigate and share the learning with those practices where observed levels are *higher* than expected. This results in improved quality of care and lower costs. In addition, by identifying and quantifying low coding levels, practices can be supported in improving their data quality. Accurate clinical coding is increasingly important in physician records for clinical audit, care planning and decision support, as well as practice performance assessment.

## A21 Population grouper decision support for healthcare and policy decisions

### Greg Zinck (gzinck@cihi.ca)

#### Case Mix, Canadian Institute for Health Information (CIHI), Ottawa, Ontario, Canada

**Background**

Using population segmentation to divide a population into health status groups can provide significant insight into healthcare needs. In Canada, many provinces have used different approaches for segmentation. The Canadian Institute for Health Information (CIHI) recently developed a population grouping methodology intended to fill the need for a common, national approach. CIHI’s innovative methodology has many potential applications across the health system for clinicians, health regions, health system funders, and researchers. This presentation showcased how it can be used for evidence-based policy decisions and health analytics.

**Methods and materials**

Population segmentation approaches describe each person in the population in terms of their significant diseases and health conditions. CIHI’s new national population grouping methodology can be applied across Canadian populations to measure their current burden of health conditions and expected resource needs in the following year. CIHI’s methodology identifies approximately 225 health conditions (e.g. diabetes, AMI, Alzheimer's, migraines), combining them with the person's functional ability and socioeconomic status to generate a set of resource indicators for each person.

**Results**

CIHI's innovative methodology project provided the first national health status population segmentation approach that can be applied across the Canadian healthcare system. The population groups and generated indicators have a wide range of potential uses that can enhance analysis, policy development, and strategic planning. The person-level data can be aggregated to generate profiles and provide insight into the differences in patient morbidity between providers, disease surveillance, and risk adjustment. The CIHI grouper creates opportunities for policy makers to develop/enhance person-based funding models based on estimates of each person's predicted cost across multiple health sectors over the next year.

**Conclusions**

CIHI's population grouping methodology is designed specifically for the Canadian healthcare system. Its applications include disease monitoring, population segmentation, risk adjustment, and funding. It has a wide range of applications for clinicians, health regions, health system funders, and researchers. CIHI’s methodology enables health system decision-makers can make more informed decisions. Its clinical profiles and resource indicators can be studied in different ways to target specific questions, such as: How do these populations compare after adjusting for morbidity? Who are likely to become high users? Is the burden of disease worsening over time? Do regions have the resources available to best serve their populations? These and other policy topics and research questions can be better understood through CIHI's population grouping methodology.

## A22 Engaging clinicians and managers early on Hospital Acquired Complications (HAC): results of a novel approach developed in Australia and Ireland to improve the quality of coded data

### Ozren Tosic, Mary E Black, Paul O'Connor

#### Pavilion Health Pty, Kirribilli, New South Wales, Australia

##### **Correspondence:** Mary E Black (maryethnablack@me.com)

**Background**

Funders are seeking to improve patients’ quality of care by penalizing hospitals for Hospital Acquired Complications (HACs). To this end, the Australian Quality and Safety Council developed a set of 38 agreed-upon clinical HACs. Coded healthcare data vary in quality, however, and can contain both false positives and false negatives about adverse events. There is also usually a considerable time-lag between data generation and hospitals being notified about funding decisions based on the data. Additionally, clinicians may not be included in this information loop, and use entirely parallel different systems than those used by hospital administrations to improve quality of care. Finally, the best Australian hospitals may appear to have a high number of HACs because they have the best systems for recording these conditions.

**Methods and materials**

A set of safety and quality data indicators based on HACs was developed and used to review more than 30 million episodes of care and identify the level of HAC under-reporting. Data were compared between peer facilities, adjusted for patient age and procedures performed. A tool involving alerts, dashboards, and regular reports was created and used by clinicians and managers to improve quality before the data were entered into official reporting systems.

Hospitals were organized into peer groups based on case-mix and specialities, and three measures were identified to rank hospitals:

A. Low reported HACs compared to peers;
B. High data quality underpinning HACs, based on the tool;
C. High level of data specificity, based on coding details (i.e., limited use of “other” and “unspecified” codes).

**Results**

Three categories of hospitals were identified by this analysis:

Category 1: All three measures are good;

Category 2: Measure A is good but B and C are poor; the facility is under-reporting HACs;

Category 3: All three measures are poor; the facility is not doing as well as it perceives with respect to HACs.

**Conclusions**

Sharing these data early with clinicians, managers, and coding teams enabled them to query and improve data early in the data cycle. Facilities were able to correct under-reporting, improve data quality; and identify patterns across individuals, teams, and organizations. Clinicians and managers will have greater confidence in data-driven rewards and penalties if they ensure their data are accurate, and are much more likely to use these data in constructive ways. This not only helps make HAC data more accurate but also improves quality of clinical care.

## A23 Clinical management in an activity-based funding environment: advancing value-based healthcare

### Andrew Blanch, Michael Lewczuk, Stuart Bowhay

#### Children's Health Queensland, Queensland, Australia

##### **Correspondence:** Stuart Bowhay (Stuart.Bowhay@health.qld.gov.au)

**Background**

Advancing value-based health care is a key goal to continue developing sustainable services and delivering the best possible outcomes for children. The activity-based funding environment has led Children’s Health Queensland (CHQ) to actively engage clinicians in identifying and addressing aspects of healthcare that balance cost-savings and clinical outcomes. Every aspect of care should add value to the patient’s journey throughout the hospital stay. CHQ had reviewed high-cost pathology investigations, since they are common in aiding diagnosis, treatment, and intervention decisions. New challenges regarding pathology costs arose after the opening of the new Lady Cilento Children's Hospital (LCCH).

**Methods and materials**

Lead clinicians in the Emergency Department (ED) developed a strategy to identify potential savings and ways to engage clinical teams to improve budgets, patient satisfaction, and quality of care. Respiratory Polymerase Chain Reaction (PCR) was identified as the ED’s largest expenditure among individual tests.

Because PCR is an invasive procedure that is often unpleasant for the patients, it also requires nursing time and resources. Clinician engagement revealed that PCR had questionable impact on decision-making, diagnosis, clinical outcomes, and prevention. An audit that examined admitted patients’ results and outcomes indicated that almost all patients who received the PCR test did so to meet due to CHQ’s practices for identifying single-room requirements, which is not an issue at LCCH. Further consultation across multiple clinical areas confirmed the indications for PCR testing had no impact on emergency decision-making.

**Results**

**Fig. 1 (abstract A23).**
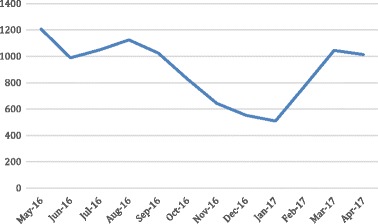
ED attendances for respiratory conditions

**Fig. 2 (abstract A23).**
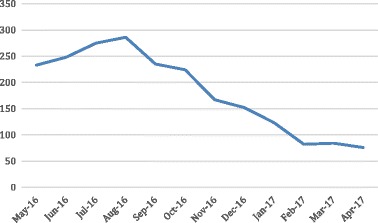
Number of Respiratory PCR tests

Clinicians were educated on the cost implications and opportunity to provide higher-value services both financially and in terms of patients’ experience. Specific criteria for using PCR tests were developed in collaboration with respiratory and infection control specialists and medical and nursing teams. These criteria empowered the nursing team to ensure indications were met before PCR testing was performed. Staff worked together and across finance, business management, and clinical care teams to analyze PCR test reason, cost, and rationalization. As a result, the numbers of Respiratory PCR tests ordered and invasive procedures provided have declined, while patient satisfaction and budgets in the ED and other departments have improved. Figure 1 shows ED attendances for respiratory conditions remained relatively stable, reducing in Spring/Summer (September–January). Figure 2 shows declines in the number of Respiratory PCR tests. Table 1 shows reductions in tests and costs.

**Conclusions**

The strategies to address and improve funding environments are transferrable to all healthcare areas to ensure that care is efficient, cost-effective, and improves patient outcomes and experiences.

**Table 1 (abstract A23).** Review of ED Respiratory PCR testing

	May-16	Jun-16	Jul-16	Aug-16	Sep-16	Oct-16	Nov-16	Dec-16	Jan-17	Feb-17	Mar-17	Apr-17
PCR Tests Undertaken	233	248	275	286	235	224	167	152	123	82	84	76
Respiratory ED Attendance	1 207	988	1 049	1 124	1 024	826	643	552	509	774	1 045	1 013
Respiratory PCR per Attendance	0,19	0,25	0,26	0,25	0,23	0,27	0,26	0,28	0,24	0,11	0,08	0,08
Respiratory PCR Costs ($)	13 626	14 503	15 472	16 090	13 221	12 602	9 395	8 552	6 920	4 613	4 726	4 726
		May-Oct 16			Nov 16-Apr 17			Variance			% Variance	
PCR Tests Undertaken		1 501			684			(817)			(54,43%)	
Respiratory ED Attendance		6 218			4 536			(1682)			(27,05%)	
Respiratory PCR per Attendance		0,24			0,15			(0,09)			(37,53%)	
Respiratory PCR Costs ($)		85 514			38 482			($47 032)			(55,00%)	

## A24 Development of the Australian Non-Admitted Care Classification (ANACC) system: results from a targeted costing study

### Divyani Bhatnagar, Jaclyn Chan, Sean Heng, Caroline Coevoet

#### Independent Hospital Pricing Authority (IHPA), Sydney, New South Wales, Australia

##### **Correspondence:** Jaclyn Chan (jaclyn.chan@ihpa.gov.au)

**Background**

The Independent Hospital Pricing Authority (IHPA) is developing the Australian Non-Admitted Care Classification (ANACC) system to replace the “Tier-2” clinic-based system. The ANACC system will be an evidence-based classification using patient-centered variables for non-admitted care services. This paper describes a component of IHPA’s statistical work in 2016 to assess the availability and quality of existing non-admitted care data in Australian national collections and cost drivers for use in developing the classification.

**Methods and Materials**

To date, two datasets have been used utilized to develop ANACC:

1. The 2013 Non-Admitted Costing Study dataset: 460,889 costed service events from six states and territories. After trimming to remove unusual costs, 445,248 records remained.
2. The 2013-2014 Australian National Hospital Cost Data Collection dataset for non- admitted care.

**Results**

A valid diagnosis was recorded for 297,030 of the costing study service events. The majority of these used a “Factors influencing health status and contact with the health service” diagnosis code, which suggested “diagnosis,” as used in admitted patient care, may be less relevant for non-admitted care. The reason for attendance/presenting problem may be more suitable in ANACC.

The most common procedure was “Non-invasive cognitive and other interventions,” reflecting that the majority of non-admitted care relates to consultations and advice. Nervous system procedures were 133% more expensive, compared to the rest of the cohort, but only represented 85 service events. Therefore, selected procedures may be more relevant in ANACC.

Age alone did not appear to be a driving factor for resource consumption, although there were important cost discrepancies with age in interaction with other patient profiles. Service events for patients aged 56+ were 11% more expensive than younger cohorts. Age may need to be a classification variable for some diagnoses and/or classes.

The 445,248 service events in the costing study dataset were bundled to 216,161 patients, of whom 64.4% received only one service event during the study. Patients receiving radiation treatment and mental health services were likely to have multiple visits for the same diagnosis; hence, some patients may be better suited to an episode-based unit of count.

There was a clear trend between increased resource consumption and a service event’s multidisciplinary status.

**Conclusions**

The data analysis provided evidence for the inclusion of variables in ANACC, such as patient diagnosis, interventions, multidisciplinary status, and age. Due to data limitations, this analysis is one of several studies used by IHPA to inform the development of ANACC.

## A25 A mutually exclusive classification for a Canadian population grouping methodology: Health Profile Groups

### Yiwen Chen, Craig Homan

#### Canadian Institute for Health Information (CIHI), Ottawa, Ontario, Canada

##### **Correspondence:** Yiwen Chen (YiChen@cihi.ca)

**Background**

In 2016, the Canadian Institute for Health Information (CIHI) released version 1.0 of its population grouping methodology and software, the first to include all persons registered in Canada’s public Medicare. The methodology assesses the population over extended time periods and multiple healthcare settings; includes a case-mix classification and predictive morbidity burden indicators; and uses a mutually-exclusive classification system – Health Profile Groups (HPG). The HPG attributes one key health condition to each patient, determined through hierarchical assessment of clinical and cost factors.

**Methods and Materials**

To create the HPG, the classification’s 226 health conditions were first rolled up to form 162 branches based on clinical and cost similarities. The process ensured high volumes at the branch level while allowing further adjustments without dramatic increases to the number of terminal groups. A physician panel vetted the branches to ensure clinical meaningfulness.

A key element of the HPG was establishing a clinical/cost ranking for tagging each person’s key diagnosis. The ranking ordered the branches and provided a logic that assigned a person with multiple diagnoses to the most significant branch.

Each branch was also linked to a higher-level category, which provided divisions between chronic and acute conditions, cancers, and mental health. They also differentiated between major, moderate, and minor conditions. The categories enabled testing of all 162 branches to determine whether the presence of comorbid conditions in specific categories impacted expected resource consumption. The results indicated that 75 branches could be split between those with and without major/moderate comorbidities. This further segmentation resulted in 237 HPGs. Groups were also created for health system users without health conditions and non-users, for a final 239 HPGs.

Cost weights were produced for each of the HPGs to indicate their projected health resource consumption relative to the population’s “average person.” These cost weights reflect the population's concurrent and future morbidity burden.

**Results**

Evaluation results showed good explanatory power for the HPG, with R^2^ as high as 31% for concurrent costs, comparing favorably with similar international methodologies.

**Conclusions**

The HPG is a valuable addition to CIHI’s population grouping methodologies. It was built using the traditional principles of case-mix design: mutually exclusiveness, clinical meaningfulness, cost homogeneity, and manageable number of groups. It profiles patients to assess present healthcare resource use and predict future utilization patterns. Cost weights may be used in funding models, to set physician capitation rates and identify high-system and high-cost users.

## A26 Developing a new non-admitted care classification in a fast-evolving healthcare environment

### Caroline Coevoet (caroline.coevoet@ihpa.gov.au)

#### Independent Hospital Pricing Authority (IHPA), Sydney, New South Wales, Australia

**Background**

The Independent Hospital Pricing Authority (IHPA) is currently developing a new classification system for non-admitted healthcare to replace the current “Tier-2” clinic-based system. There have been significant shifts and developments in non-admitted care in recent years, a trend that will continue in the future. A new classification system must respond to, and anticipate, these changes. This paper gives an overview of the changing healthcare landscape and identifies the main issues for consideration in the development of the Australian Non-Admitted Health Care Classification.

**Results**

There has been a consistent view that any new long-term non-admitted care classification needs to account for the increasing complexity of patients who are seen in the non-admitted setting, particularly those with chronic conditions, multiple comorbidities, and complex psychosocial situations. It should also address the many patients who receive intensive multi-disciplinary management from a number of healthcare providers.

In addition, the new classification needs to be flexible and account for, and adapt to, newer models of care and technology; encourage the delivery of integrated care between healthcare settings, particularly between admitted and non-admitted settings; and address integration of services with primary care providers.

While there is a drive to move care out of inpatient settings, information systems generally lag behind such changes. In most Australian non-admitted settings, activity is captured through ageing appointment systems and paper-based medical records. Patient information may be disseminated across multiple systems; there is significant variability in e-referral and information systems across jurisdictions, within jurisdictions, and even within individual hospitals.

The new classification must account for trends in e-health and advances in electronic medical record use and health terminologies, such as SNOMED’s growing use across disciplines. Care delivery will continue to evolve, and care that has historically been provided in the admitted setting will continue transitioning to the non-admitted setting. The new classification will also need to overcome barriers to detect patient cohorts as they cross between the care type silos and enable longitudinal tracking as models of care change over time. These trends point to the need for interoperability of a non- admitted classification with admitted and primary care.

**Conclusions**

The new non-admitted care classification must take advantage of these advances and be relevant to them, and ensure longevity, with use updates rather than whole-scale revisions. As such, classification development will need to be informed by the opportunities of the future landscape, rather than restricted by current reporting requirements and limitations.

## A27 Evolution of the French classification for non-acute activity

### Nicolas Dapzol, Meriem Said, Fabrice Elegbede, Axelle Menu, Marie-Caroline Clement, Joëlle Dubois

#### Technical Agency for Hospital Information, Lyon, France

##### **Correspondence:** Nicolas Dapzol (nicolas.dapzol@atih.sante.fr)

**Background**

A new classification (Economic and Medical Group, or GME) was introduced in France in 2013, as the first step in implementing activity-based funding for non-acute care inpatient stays. The GME classification has two hierarchical levels addressing the principal and associated diagnoses, and a third level combining all other information (i.e., disability, procedures, age, etc.). French non-acute care hospitals are divided into specialized vs. non-specialized hospitals that combine technical rehabilitation platforms and post-acute care units.

Physicians and managers report that the GME is hard to interpret and that, due to its structure, the case-mix is hard to analyze with respect to the patient complexity of stay and the rehabilitation received. The classification was updated to address these challenges; it now describes the patient’s main pathology, complexity for a fixed pathology, and rehabilitation received for fixed pathology and complexity.

**Materials and methods**

The work was conducted on the national public and private databases of patient hospitalizations from 2014 to 2016 (around 4,000,000 stays) and a cost study (around 83,000 stays). Medical groups, the classification’s first level, remained unchanged from the first GME classification. Subgroups were introduced to describe pediatric activity and some specific rehabilitation services. Complexity levels were introduced to describe the economic weight given a specific pathology based on secondary diagnoses, disability, age, and surgical anteriority. This synthesis was made by optimizing the R-squared (R^2^ ) of the length of stay (LOS) and costs. Rehabilitation groups were used to describe the amount of rehabilitation received given the pathology and complexity level, which also provide macro-economic information.

**Results**

The updated classification is comprised of 108 medical groups and sub-groups, each divided into three complexity levels and two rehabilitation levels, for a total of 648 inpatient hospitalization groups, and 216 day hospitalization groups. For inpatient hospitalization, the LOS R^2^ is 14.4% and the cost of stay R^2^ is 30.0%. Classification biases were tested on specific populations (i.e., elderly patients, dependent populations, etc.) and different establishment types.

**Conclusions**

The updated classification attempted to describe the pathology, complexity of stay, and rehabilitation provided to patients in French non-acute hospitals. The update included a merger of information that had previously been accounted for separately in the case-mix system. Some stakeholders fear, however, that this merger of diverse information may interfere with the coding process and make the classification less understandable. The classification process is still evolving.

## A28 Reviewing established grouping parameters: comorbidity level in Case Mix Group Plus (CMG+)

### Minh Duong-Hua, Craig Homan, Jeff Hatcher

#### Canadian Institute for Health Information (CIHI), Ottawa, Ontario, Canada

##### **Correspondence:** Minh Duong-Hua (mduong-hua@cihi.ca)

**Background**

The Canadian Institute for Health Information (CIHI) has a number of established grouping methodologies under its jurisdiction, including Case Mix Group Plus (CMG+), an inpatient grouping methodology. Part of CIHI’s mandate with these methodologies is to ensure that previously-established parameters remain effective and accurate going forward. In 2017, CIHI investigated CMG+’s Comorbidity Level (CL), a significant factor that contributes to the calculation of two CMG+ resource indicators: Expected Length-of-Stay (ELOS) and Resource Intensive Weights (RIW).

CL is determined by specific comorbidities’ presence, absence, and combination. Comorbidities are identified through regression models that perform impact assessments for each diagnosis in each Major Clinical Category (MCC). The result is a body-system-specific list of diagnoses with an associated comorbidity “factor.” Individual factors are used to derive the CL assignment.

Historically, comorbidities only appeared on the MCC-specific list when they showed a minimum 25% impact on costs and/or ELOS, to ensure only significant diagnoses were included in the model (i.e., threshold set at 1.25). Thresholds other than 25% were tested to evaluate the impact on resource indicators, and the methodology’s explanatory power, which is important given changing medical practices and cost data.

**Materials and methods**

The data used for the derivation of comorbidity factor values for CMG+ 2015 were comprised of Canadian Patient Cost Data for fiscal year 2009-2010, 2010-2011, and 2011-2012. The study analyzed several alternative adjustment factors thresholds (e.g., 1.20, 1.30, 1.40) and corresponding alternative CL ranges. The study assessed the impact on case volume within each CL assigned and the changes in final RIW.

**Results**

Each different threshold resulted in varying comorbidity lists, with 1.20 having the largest and 1.40 the smallest, as expected. The final comorbidity list and case volume within any CL may dramatically change from one threshold to another within each MCC. RIW shift showed 90% of cases were within 10% of the original RIW, although there were larger shifts in the 1.40 group. The “goodness of fit” statistics for the RIW model performed slightly better using the current methodology; R^2^ ranged from .7948 to 8033, compared to R^2^ of 0.8081 for the current CL threshold of 1.25.

**Conclusions**

The analysis did not indicate a reason to change the current CL 1.25 adjustment factor value, but was still important to complete. Rechecking factors in all models is always a vital part of on-going evolution for groupers to maintain their accuracy and applicability in a changing healthcare landscape.

## A29 Benchmarking variation in coding across hospitals in Canada: A data surveillance approach

### Lori Kirby (lkirby@cihi.ca)

#### Canadian Institute for Health Information (CIHI), Ontario, Canada

**Background**

Ensuring the use of high-quality data for funding decisions is paramount to the success of any reform initiative. While funding initiatives can influence data quality positively in terms of attention and resources directed toward improvement, there is also the risk of data being manipulated to improve outcomes. Ontario's funding formula uses data from several national databases housed at the Canadian Institute for Health Information (CIHI). These databases provide information on patient activity and clinical status across the care continuum. CIHI collaborated with the Ontario Ministry of Health and Long-Term Care (MOHLTC) and Ontario Hospital Association (OHA) to monitor the quality of these data and assess any influence upon coding behavior.

**Methods and materials**

A Data Surveillance Program (DSP) was implemented in Ontario to ensure acute care hospitals report high-quality data to the Discharge Abstract Database (DAD), which is used for health system planning and funding. The DSP includes enhanced data quality reports that dig into data using modeling techniques (often associated with “big data”) to identify anomalies. The models identify what “typical” clinical coding patterns are for key patient groups, so that outliers can be identified, where patients do not fit the expected pattern. The Ontario’s funding reform includes Quality Based Procedures (QBPs), through which hospitals are reimbursed according to types and quantities of patients treated. QBPs are specific groups of patient services that enable healthcare providers to share best practices and achieve better quality and system efficiencies.

**Results**

This analysis focused on Ontario’s QBP populations to assess whether jurisdictions where QBPs do not affect funding observe similar patterns around overall volume, length of stay, and Case Mix Index (CMI). Using the DSP, several Ontario hospitals were identified with coding practices that appear to differ from their peers. CIHI will further investigate these coding variations observed in Ontario by benchmarking against other Canadian jurisdictions using the same big data techniques.

**Conclusions**

The DSP uses a series of standardized scores identifying whether a hospital is an outlier for certain indicators. These scores allow hospitals to compare themselves against their peers within the province. Extending the analysis to benchmark similar hospitals on a national scale could indicate whether the coding variations seen in Ontario are a result of the introduction of QBPs to the funding formula or a natural variation across the country.

## A30 Reporting the efficiency of Australia’s public hospitals on the MyHospitals website

### Anna O'Mahony, Eve Kelly, Anika Merkley

#### Australian Institute of Health and Welfare, Canberra, Australian Capital Territory, Australia

##### **Correspondence:** Anika Merkley (anika.merkley@aihw.gov.au)

**Background**

The Australian Institute of Health and Welfare (AIHW) is responsible for reporting on hospital performance, as identified by the Council of Australian Government’s Performance and Accountability Framework (PAF). The AIHW reports PAF performance indicators via its MyHospitals website. The MyHospitals website was established in 2010 to provide consumers, clinicians, service providers, and policymakers with access to nationally consistent, locally relevant, and comparable information. It is also designed to support improvements in the healthcare system through increased transparency and accountability.

**Materials and methods**

The MyHospitals website supports a range of tools to engage and inform its broad audience and stakeholders. Users have access to data, reports, and tools including interactive tables, charts, and Excel downloads. Key findings are released in detailed, downloadable reports. The upcoming AIHW release “*Costs of acute admitted patients in public hospitals from 2012–13 to 2014–15”* will report the relative efficiency of Australia’s largest public hospitals over time. It will build upon two previous reports of the same name published in 2015 and 2016. The previous “*Costs of acute admitted patients in public hospitals from 2011–12 to 2013–14”* report (released in 2016) displayed a three-year time series, and highlighted eight major metropolitan public hospitals that had improved their efficiency during this timeframe.

**Results**

The MyHospitals website reporting of the efficiency of Australia’s public hospitals presents the cost per national weighted activity unit measure, using the most recently available hospital cost data. This measure determines the cost of a notional “average” service at individual hospitals, and allows for comparisons between hospitals and over time. This narrative will be enhanced through the annual release of updated cost data, and development and publication of a new Relative Stay Index measure. The new Relative Stay Index measure will allow analysis of how quickly hospitals discharge similar types of patients, compared to their peers.

**Conclusions**

Reporting hospital efficiency provides valuable insights into variations in the relative efficiency and performance over time of Australia’s largest public hospitals. The MyHospitals website provides nationally consistent, locally relevant performance information, increases transparency, improves accountability, and informs decision-making.

## A31 Australian emergency care classification development

### Jim Pearse^1^, Deniza Mazevska^1^, Joel Tuccia^1^, Zachary Davies^1^, Aaron Balm^2^

#### ^1^Health Policy Analysis, Pty, Ltd., Sydney, New South Wales, Australia; ^2^Independent Hospital Pricing Authority, Sydney, New South Wales, Australia

##### **Correspondence:** Jim Pearse (jpearse@healthpolicy.com.au)

**Background**

In 2013, the Independent Hospital Pricing Authority (IHPA) commissioned work to recommend options for classifying public hospital emergency care. The review concluded that the Urgency Related Groups and Urgency Disposition Groups classification systems currently used in Australia were unsuitable for continued use, due to reliance on “urgency” as the key classification variable. There was also an interest in moving to a classification based on diagnosis, with better complexity markers and enhanced clinical utility. Therefore, IHPA commissioned the development of a new classification system for emergency care.

**Materials and methods**

A costing study was conducted to obtain more-detailed activity and cost data than was available through routine national collections. The study included 10 Emergency Departments (EDs) across Australia, representing different sizes and roles, which collected four weeks of data between April-June 2016. Classification structure options are being explored and tested based on these data, including:

- A first level that segments ED visits from other presentation types that do not require further splitting (e.g. patients who did not wait to be seen by a clinician);
- A second level that partitions ED visits into groups of diagnoses that make sense clinically, representing similar care processes;
- A third level that further splits classes based on complexity, where required.

**Results**

Variables being considered as complexity markers include additional diagnoses, diagnosis “modifiers” (i.e., conditions/states contributing to patients being more resource-intensive than expected given their presenting condition, e.g. heightened distress, confusion, or agitation), procedures, investigations, urgency, age, and arrival mode. Some of these data are routinely collected; others were collected specifically for the costing study. Therefore, the best complexity markers may not be immediately available. There is a need for an immediately implementable classification, and medium- to long-term solutions requiring further data development, to create new elements for classification.

**Conclusions**

Key classification challenges include:

- Ensuring suitability for specific subpopulations (e.g., pediatrics, mental health);
- Identifying cost variability that is best addressed by classification versus other mechanisms such as pricing;
- Accurately reflecting resource use, while minimizing perverse incentives (e.g. extent of use of procedures to define classes).

The classification system must perform consistently when tested against data not used to generate the classes, to ensure the classification’s predictive power is not overstated. To address this, cross-validation is used, where models are developed using one data partition (the “training data”) and tested against another partition. The Australian emergency care classification is anticipated for implementation in 2019-2020.

## A32 Revisiting inpatient rehabilitation case-mix and funding models in Ontario, Canada: lessons learned

### Kristen Pitzul^1^, Emitis Moshirzadeh^1^, Jan Walker^2^, Kevin Yu^3^, Sandro Serino^1^, Imtiaz Daniel^1^

#### ^1^Ontario Hospital Association, Toronto, Ontario, Canada; ^2^WestPark Healthcare, Toronto, Ontario, Canada; ^3^Ontario Ministry of Health and Long-Term Care, Toronto, Ontario, Canada

##### **Correspondence:** Kristen Pitzul (kpitzul@oha.com)

**Background**

Activity-based funding models (ABFMs) are well-established in the acute care period, due to their importance in bundled payment systems. ABFMs’ development and implementation in post-acute settings (like rehabilitative care) have, however, posed unique challenges in healthcare systems worldwide. Many jurisdictions are just beginning to focus on refining these ABFM models. The purpose of this study was to review rehabilitative care ABFM models in jurisdictions worldwide.

**Materials and methods**

In Ontario, medically-necessary care is funded by a single public payer; total expenses for fiscal year 2015-2016, for all rehabilitation beds, was $585 million (Canadian dollars). A group of experts was formed to assess Ontario’s current case-mix grouper and ABFM for inpatient rehabilitation (IPR). Ontario’s current IPR case-mix includes 83 patient groups based on functional measures and patient age; funding is based on each group’s cost weight with modified length of stay outliers and other adjustments (e.g., hospital teaching status).

A literature review and semi-structured interviews were conducted to gain insight into other jurisdictions’ case-mix groupers and funding models. An initial review was conducted of English-language report summaries (i.e., grey literature) and peer-reviewed literature published from the year 2000 onwards. A list of content experts from other jurisdictions was generated from this initial literature review and authors’ existing contacts. A random sample of these experts was contacted for a one-hour interview. Additional literature suggested by the interviewed content experts was also included. Literature search and interview results were summarized together.

**Results**

The literature review yielded over 2,000 articles. After searching titles and abstracts, fewer than 100 articles and reports were deemed to be relevant. Content experts from Australia, United States, United Kingdom, Norway, Denmark, Estonia, Germany, Finland, Iceland, France, and Sweden were contacted. Interviews were conducted with experts from six countries, and information exchanged with another four experts.

**Conclusions**

Results suggest most jurisdictions have developed an IPR case-mix grouper, but many struggle with implementation of ABFM. Lessons can be gleaned from the United Kingdom, Australia, and the United States (including the lack of diagnosis-related groups and weighting of motor functional items), but many jurisdictions struggle with challenges like those experienced in Ontario (including data availability). Results can foster collaborations between jurisdictions to maximize information exchange and address challenges for IPR case-mix and ABFM.

## A33 CIHI's population grouping methodology: beyond predicting cost

### Yvonne Rosehart, Jeff Hatcher

#### Canadian Institute for Health Information (CIHI), Ottawa, Ontario, Canada

##### **Correspondence:** Yvonne Rosehart (yrosehart@cihi.ca)

**Background**

The Canadian Institute for Health Information (CIHI) recently released version 1 of its population grouping methodology. The grouper, which creates health profiles and assigns associated cost weights for all individuals with a valid health card number, also estimates future use of select healthcare system resources. Based on health conditions, the population grouper predicts an individual’s number of visits to a family medicine physician and to an Emergency Department (ED) in the upcoming year. For those ages 65 and older, it predicts the likelihood of admission to a long-term care (LTC) facility within the next year.

**Methods and materials**

CIHI used age, sex, the 226 health conditions, and the most influential two-way health condition interactions as predictor variables. For individuals with no health conditions, or who did not access the healthcare system in the study period, CIHI used age and sex as predictor variables.

Using the generalized linear models approach, CIHI developed separate models for the three indicators. To predict the number of visits to a family physician, CIHI developed a linear regression model assuming a normal distribution. To predict the number of ED visits, a two-stage model was developed: Stage 1 used logistic regression to calculate the probability of visiting the ED, Stage 2 used an ordinary least squares linear regression to predict the number of ED visits. The stages’ outputs were multiplied to calculate predicted number of ED visits. To predict LTC admissions, a logistic regression model was used; given the rarity of entering LTC, a choice-based sampling technique was utilized to improve model performance.

**Results**

For each model, the final regression technique chosen maximized the overall predictive power and goodness-of-fit. The goal was to predict individuals’ expected health system utilization, which were then segmented into population cohorts. Evaluation results showed good explanatory power for each model, with R^2^ of 28% and 26% for models predicting primary care and ED visits, respectively; and a C-statistic = 0.90 for the model predicting LTC admission.

**Conclusions**:

The three predictive indicators provide policymakers and planners with insight for use in capacity and human resource planning, communication with selected patient cohorts (e.g. high ED users or those more likely to need LTC), and analysis of service delivery models. Including these predictive indicators in electronic medical records can provide healthcare professionals with additional information at the point of treatment, which might help to inform individual patient care plans.

## A34 Measurement of health organization performance across multiple sector services: a population health analytic perspective

### Stephen Sutch^1^, Alan Thompson^2^, Chad Abrams^3^

#### ^1^Johns Hopkins HealthCare Solutions, Baltimore, Maryland, United States; ^2^Johns Hopkins HealthCare Solutions, Romsey, England, United Kingdom; ^3^Johns Hopkins HealthCare Solutions, Baltimore, Maryland, United States

##### **Correspondence:** Stephen Sutch (ssutch1@jhu.edu)

**Background**

When measuring health organizations’ activities and performance, it is important to consider the case-mix, technical efficiencies, inter-dependencies between organizations, and whether organizations provide different services. The measurement of primary care practices and their inter-dependency with secondary care services is an example of the challenges facing proper measurement and interpretation of outcomes. This study measured the effects of substitution and overlap of primary and secondary care activities, and provided a population health analytic approach to this area.

**Methods and materials**

Primary and secondary care data from multiple studies in various countries were combined to create population databases. Case-mix measurements were made using the Johns Hopkins Adjusted Clinical Groups (ACG) System, to control for case-mix complexity and multimorbidity. The populations’ distribution was analyzed with respect to the share of activity and costs between primary, outpatient, and inpatient services. The triple aim reporting framework suggested by Seow [1] was adapted to examine temporal changes in cost distribution by health sector and different organization models.

**Results**

Analysis of total costs across all sectors (i.e., inpatient, outpatient, emergency care, primary care) showed increased costs associated with higher complexity and multimorbidity patients. Distribution of utilization and cost varied according to patient complexity and organization. For example, the proportion of spend on primary care services was generally higher for lower-complexity patients, with the proportion decreasing with increased complexity. Increased spend on hospital services was seen as complexity increased. Regional analyses highlighted that proportions of spend were different even when case-mix complexity was held constant. The population-level analyses showed different results with respect to cost efficiency compared to individual sector results, for example, perceived low-cost organizations achieved these results due to apparent cost and utilization shifts to other sectors.

**Conclusions**

Measuring true performance is challenging when sectors of care exist across organizations, and where healthcare delivery is divided across silos. Introduction of integrated health services (i.e., accountable care organizations, local health community, population health programs, etc.) requires an adequate framework in order to assess cost and improvements to health quality, and attribute any improvements occurring across multiple organizations. Case-mix adjustment is an important consideration in measuring healthcare organizations. To reduce confounding, the inter- and intra-organizational dynamics and dependencies need to be considered.

**References**

1. Seow HY, Sibley LM. Developing a dashboard to help measure and achieve the triple aim: a population-based cohort study. BMC Health Services Research 2014; 14:363.

